# Transcriptomic analysis reveals the mechanism of thermosensitive genic male sterility (TGMS) of *Brassica napus* under the high temperature inducement

**DOI:** 10.1186/s12864-019-6008-3

**Published:** 2019-08-13

**Authors:** Xin Tang, You-Jin Hao, Jun-Xing Lu, Geng Lu, Tao Zhang

**Affiliations:** Collage of Life Sciences, Chongqing Normal University, Chongqing, 401331 China

**Keywords:** *Brassica napus*, Thermo-sensitive genic male sterility, Differential gene expression, Hormone signaling, Transcription factor

## Abstract

**Background:**

The thermo-sensitive genic male sterility (TGMS) of *Brassica napus* facilitates reproductive researches and hybrid seed production. Considering the complexity and little information about the molecular mechanism involved in *B. napus* TGMS, comparative transcriptomic analyses were peroformed for the sterile (160S-MS) and fertile (160S-MF) flowers to identify potential crucial genes and pathways associated with TGMS.

**Results:**

In total, RNA-seq analysis showed that 2202 genes (561 up-regulated and 1641 down-regulated) were significantly differentially expressed in the fertile flowers of 160S-MF at 25 °C when compared the sterile flower of 160S-MS at 15 °C. Detailed analysis revealed that expression changes in genes encoding heat shock proteins, antioxidant, skeleton protein, GTPase and calmodulin might be involved in TGMS of *B. napus*. Moreover, gene expression of some key members in plant hormone signaling pathways, such as auxin, gibberellins, jasmonic acid, abscisic acid, brassinosteroid signalings, were significantly surppressed in the flowers of 160S, suggesting that these genes might be involved in the regulation in *B. napus* TGMS. Here, we also found that transcription factor MADS, NFY, HSF, MYB/C and WRKY might play a crucial role in male fertility under the high temperature condition.

**Conclusion:**

High temperature can significant affect gene expression in the flowers. The findings in the current study improve our understanding of *B. napus* TGMS at the molecular level and also provide an effective foundation for male fertility researches in other important economic crops.

**Electronic supplementary material:**

The online version of this article (10.1186/s12864-019-6008-3) contains supplementary material, which is available to authorized users.

## Background

Heterosis has been applied to increase the crop yield, alleviate the contradiction between quality and production and stress resistance. Male sterile (MS) is one of the most important method utilized in the heterosis. As a common biological phenomenon, plant MS system is also valuable for studies on anther development, organogenesis, cytoplasmic inheritance, and nucleo- cytoplasmic interactions [1–3]. Presently, MS types used in the hybrid breeding mainly include cytoplasmic male sterility (CMS), genic male sterility (GMS), self incompatibility (SI) and chemical gametocide (CG) [4–7]. CMS is characterized by the production of non-functional pollen and has been used to generate hybrids with significant heterosis in some crops. However, this sterility type is not stable in sterile line (sometimes carrying a trace of fertile pollen). Moreover, parental screening is affected by the restore line and/or the maintenance line, and also negatively affected by the cytoplasm. Although, three-line system has been applied for the hybrid seed production, a pure CMS line is difficult to be obtained. The CG method is influenced by the weather conditions and the contents of chemical gametocide agents and application time are hard to control. Therefore, the development of new male sterile materials and understanding the molecular mechanism are essential for rapeseed cultivation and other economic crops. Since the first report of photo/thermo-sensitive genic male sterility (P/TGMS) of rice 58S was reported [8], many P/TGMS lines in some plant species have been developed [9], which is characterized by the fertility that is not only genetically controlled but also affected by the temperature, photoperiod and other environmental factors. Thermo-sensitive genic male sterility (TGMS) is a special sterile system, in which anther development can be depressed under the sterile conditions and non-functional anthers are produced. While under the fertilizing conditions, the depression is totally or partially removed and functional anthers can be produced.

Great progresses have been made in understanding the molecular mechanisms of rice P/TGMS. A mutation in a short-form *RNase Z* produce a TGMS rice and currently has been used for hybrid rice production [10]. The TGMS trait of Hengnong S-1 is controlled by *tms9–1*, encoding a PHD-finger protein [11, 12]. Over-expression of UPD-glucose pyrophosphorylase 1(*Ugp-1*) in rice can produce TGMS trait under the normal temperatures and this trait can be reverted at a low temperature. In addition, many genes encoding transcription factors, members of some signal transduction pathway or proteins involved in metabolisms have been identified from low-temperature treated rice TGMS Y58S and Pei ai 64S [13]. Interestingly, methylation level is significantly higher in the sterile Pei ai 64S than in fertile Pei ai 64S [14, 15]. These results enriched our understanding of the molecular mechanisms of TGMS.

Several ecotypical TGMS rapeseed lines have been identified and applied to produce hybrids, but development and application of novel TGMS lines will enrich the utilization of the heterosis to increase yield and oil seeds quality. Moreover, it is also helpful for understanding the molecular mechanism of the male sterility. So far, numerous differentially expressed genes have been identified in flowers or anthers of *Brassica oleracea* [16, 17], *Brassica rapa* [18], *Brassica juncea* [19], and *Brassica napus* [20–22], which outlines an intricate transcriptional regulation that occurs in TGMS system when the temperature is changed. Although numerous efforts have been made in molecular dissections of the fertility of sterile materials, conversion between the fertility and sterility, and anther abortion is not well-known. Due to the complexity of TGMS, previous studies on the regulatory mechanism are far from enough in the depth and breadth. To further explore the mechanisms and promote their wider applications, it is necessary to study it more deeply and systematically.

*B. napus* is a world widely economic crop and an important source of edible oil. Our previous study showed that the temperature below 20 °C can induce the male fertility (160S-MF) of 160S, whereas the temperature above 25 °C can induce the sterility (160S-MS). The fertility rate, pod-setting ratio and seed number per pod correspondingly decreased with the increased temperature [23], suggesting that the temperature is a key factor to affect the morphology of flower and the fertility of pollens. However, the molecular regulatory mechanisms are unknown. Therefore, as the first step towards understanding the molecular mechanism of the TGMS in rapeseed, we aim to mine the sterility-related gene by RNA-seq approach, which has been widely used to identify deferentially expressed genes from two or more compared samples. In this study, the flowers of 160S-MF and 160S-MS were used to characterize gene expression patterns. Eight TGMS-related candidate genes were further validated by quantitative realtime-PCR (qPCR). In this way, this study explored the relationship between the male sterility/fertility and gene expression patterns, to obtain more insights in TGMS and offered valuable genetic information for breed improvement and the application of *B. napus*.

## Results and discussion

### Transcriptome sequencing

Two transcriptome libraries were constructed from the flowers of 160S-MF and 160S-MS. High throughput sequencing generated 57,818,154 raw reads for 160S-MF and 68,606,002 raw reads for 160S-MS. After removing the adaptor, low-quality and ambiguous nucleotides, 45,263,606 and 60,008,172 high quality reads were obtained for 160S-MF and 160S-MS, respectively. The average length of the clean reads is 98.66 bp in 160S-MF and 98.7 bp in 160S-MS (Additional file 1: Table S1). All the raw transcriptome data have been deposited at the GenBank (PRJNA. 513,109). Reads assembly produced 101,040 unigenes with 46% GC content.

All assembled unigenes were analyzed by BLAST search against the NR, Swiss-Prot, PFAM, TREMBL and KOG databases (*E* < 1e-5). The results showed that 65.35% unigenes significantly matched to the genes in TREMBL database, followed by 60.0% in NR database, 44.47% in PFAM, 35.23% in SWISS-PROT database and 10.65% in KOG database (Additional file 2: Figure S1A). Unmatched unigenes could be attributable to the short sequence reads, or might be unique to *B. napus*, or the relatively short sequences lacked conserved functional domains. The hitted homologue distribution analysis revealed that 36% unigenes was matched to the homologues in *Arabidopsis thaliana*, 28% matched to *Arabidopsis lyrata subsp. lyrata*, and 23% matched to *Capsella rubella* (Additional file 2: Figure S1B).

**Fig. 1:**
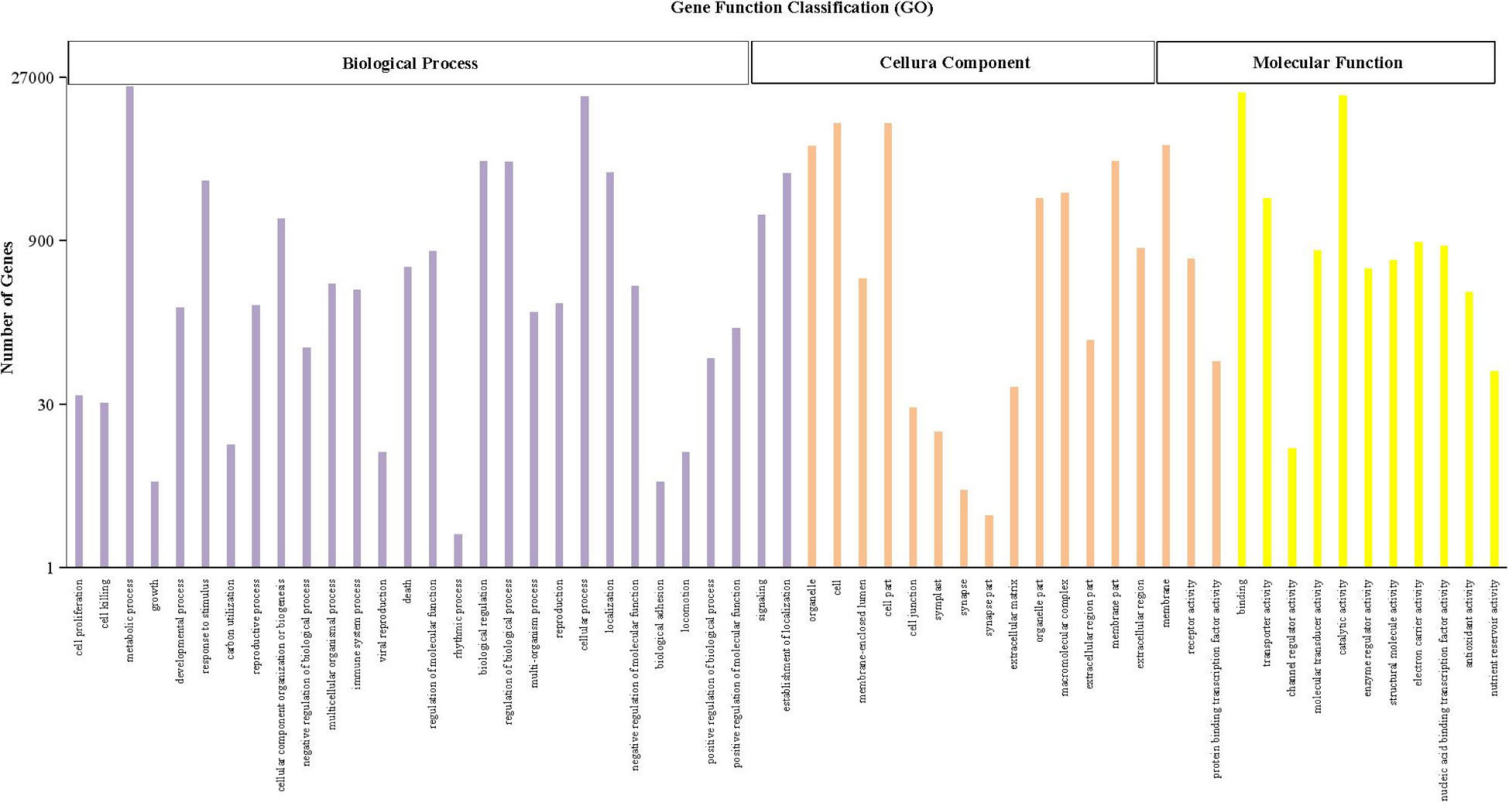
Gene function classification of all annotated unigenes by Gene Ontology (GO). The horizontal axis represents the specific GO sub-categories, and the vertical axis represents the number of unigenes

GO assignments were used to classify the gene functions using the Blast2GO. In all, 31,482 unigenes were mapped and categorized into “biological process”, “cellular component” and “biological process” (Fig. 1). In biological process category, “metabolic process” (GO:008152) presented the largest cluster, accounting for 22.0%, followed by “cellular process” (GO:009987; 18.1%) were the most abundant groups. In cell component category, “cell” (GO:005623; 10.3%) and cell apart (GO:0044464; 10.3%) were predominant. Under the molecular function category, genes related to “binding” (GO:005488; 19.3%) and “catalytic activities” (GO:003824; 18.1%) were the most highly represented. Detailed GO data were shown in Additional file 3: Table S2.

### KOG and KEGG pathway enrichment

**Fig. 2:**
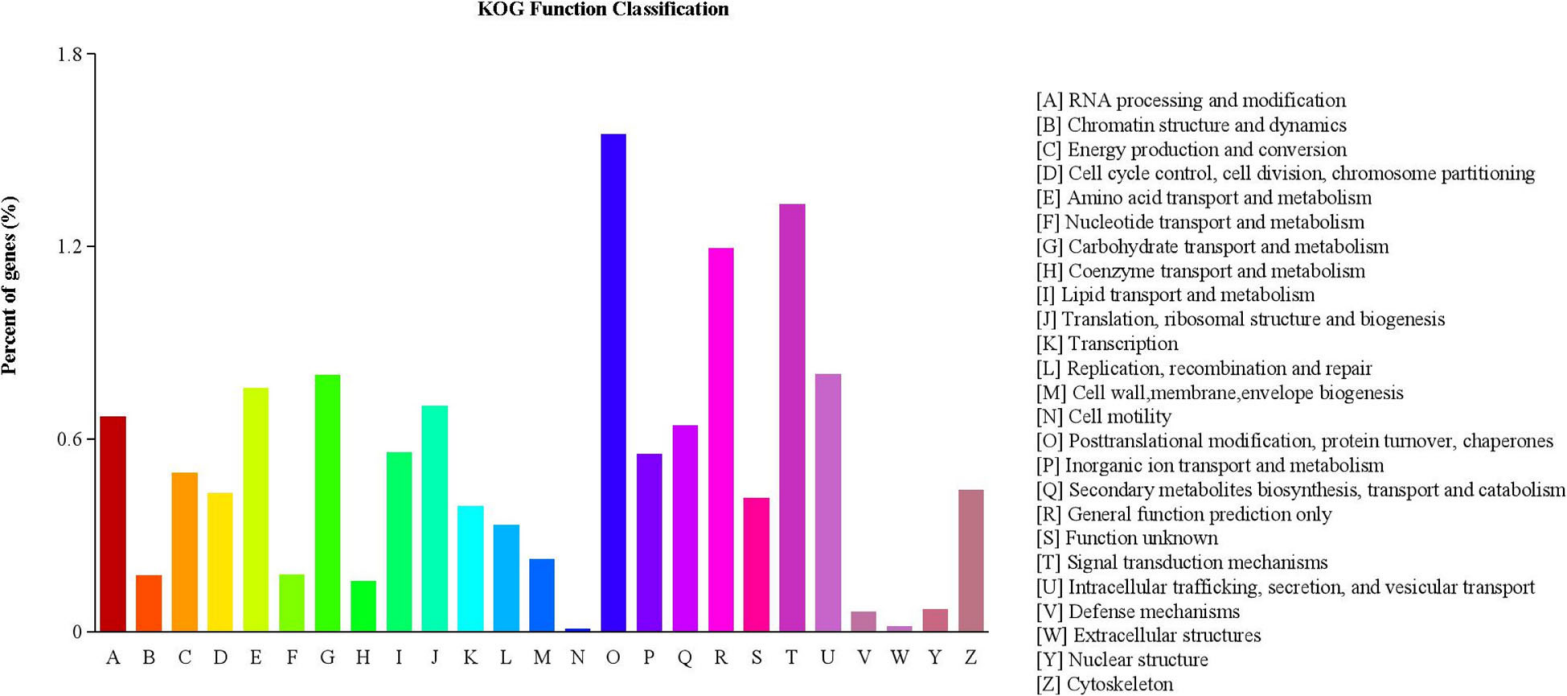
KOG functional classification of all mapped unigenes

**Fig. 3:**
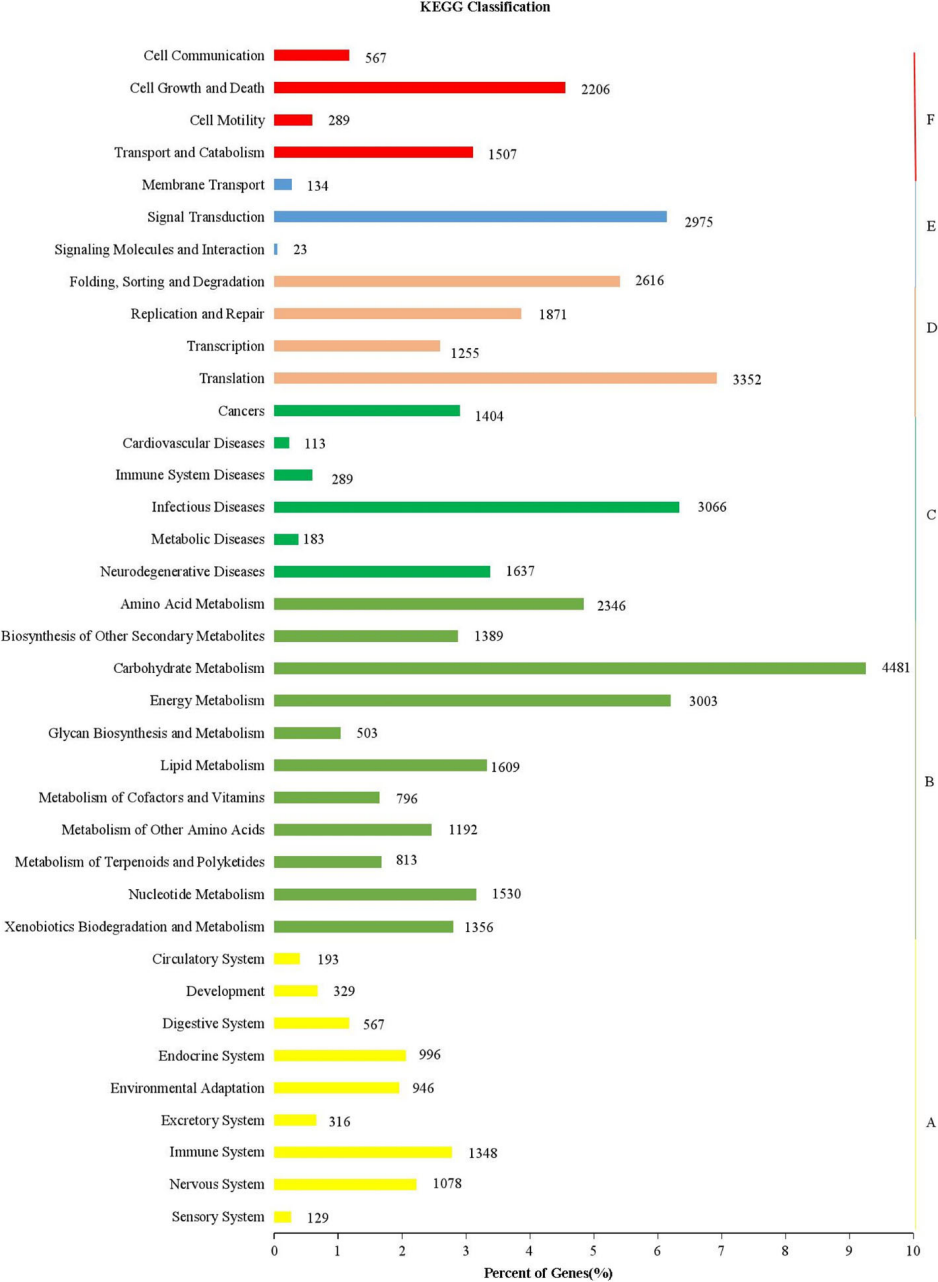
Histogram of cluster of KEGG pathways of assembled unigenes. The horizontal axis is the gene number; and vertical axis is the name of cluster of KEGG. A: Cellular processes; B: Environmental information processing; C: Genetic information processing; D: Metabolism; E: Organismal systems; and F: Cellular Processes

KOG classification indicated that 31,482 unigenes were clustered into 25 functional categories (Fig. 2). Unigenes in “post-translational modification, protein turnover, chaperones” category were the most abundant (1568 ungienes, 1.55%), followed by “signal transduction mechanisms” (1345 unigenes, 1.33%), “general function prediction only” (1207 unigenes, 1.19%), “intracellular trafficking, secretion, and vesicular transport” (811 unigenes, 0.8%) and “carbohydrate transport and metabolism” (809 unigenes, 0.80%), “nuclear structure” (73 unigenes, 0.07%), “defense mechanisms” (65 unigenes, 0.06%), “extracellular structures” (19 unigenes, 0.02%) and “cell motility” (62 unigenes, 0.009%) was the smallest group. Detailed KOG data were shown in Additional file 4: Table S3. To better understand the gene functional differences, the up- and down-regulated differentially expressed genes (DEGs) were grouped by KEGG (Fig. 3). The results revealed that 27,817 (88.4%) transcripts were assigned to 295 pathways (Additional file 5: Table S4). The top 5 pathways were associated with ribosome (ko03010, 1281 genes), plant hormone signal transduction (ko04075, 1116 genes), protein processing in endoplasmic reticulum (ko04141, 800 genes), spliceosome (ko03040, 731 genes), and starch and sucrose metabolism (ko00500, 720 genes).

### Differentially expressed genes between 160S-MF and 160S-MS

The expression levels were measured using reads per kilobase of exon model per million mapped sequence reads (RPKM). RPKM values for each gene in 160S-MS and 160S-MF were compared to identify DEGs and 2202 DEGs (561 up-regulated and 1641 down-regulated) were produced.

To further determine how these DEGs were involved in the biological processes, KEGG analysis was performed and 204 pathways were enriched (Additional file 6: Table S5). For up-regulated genes in the flowers of 160S-MS, the top 5 pathways were associated with plant hormone signal transduction (ko04075, 13 genes), glyoxylate and dicarboxylate metabolism (ko00630, 12 genes), protein processing in endoplasmic reticulum (ko04141, 11 genes), carbon fixation in photosynthetic organisms (ko00710, 9 genes), and starch and spliceosome (ko03040, 8 genes). For the down-regulated genes in 160S-MS flowers, the top 5 pathway mainly associated with starch and sucrose metabolism (ko00500, 31 genes), regulation of actin cytoskeleton (ko04810, 26 genes), pentose and glucuronate interconversions (ko00040, 25 genes), Fc gamma R-mediated phagocytosis (ko04666, 21 genes) and shigellosis (ko05131, 16 genes).

In this study, it was worthy to be mentioned that unigenes in the pathways responsible for thermo-sensitive genic male sterility included oocyte meiosis, plant hormone signal transduction, RNA degradation, ubiquitin mediated proteolysis, protein processing in endoplasmic reticulum, spliceosome, and mRNA surveillance pathway may provide valuable resource for the identification of unique genes involved in TGMS.

### Validation of differentially expressed genes

**Fig. 4:**
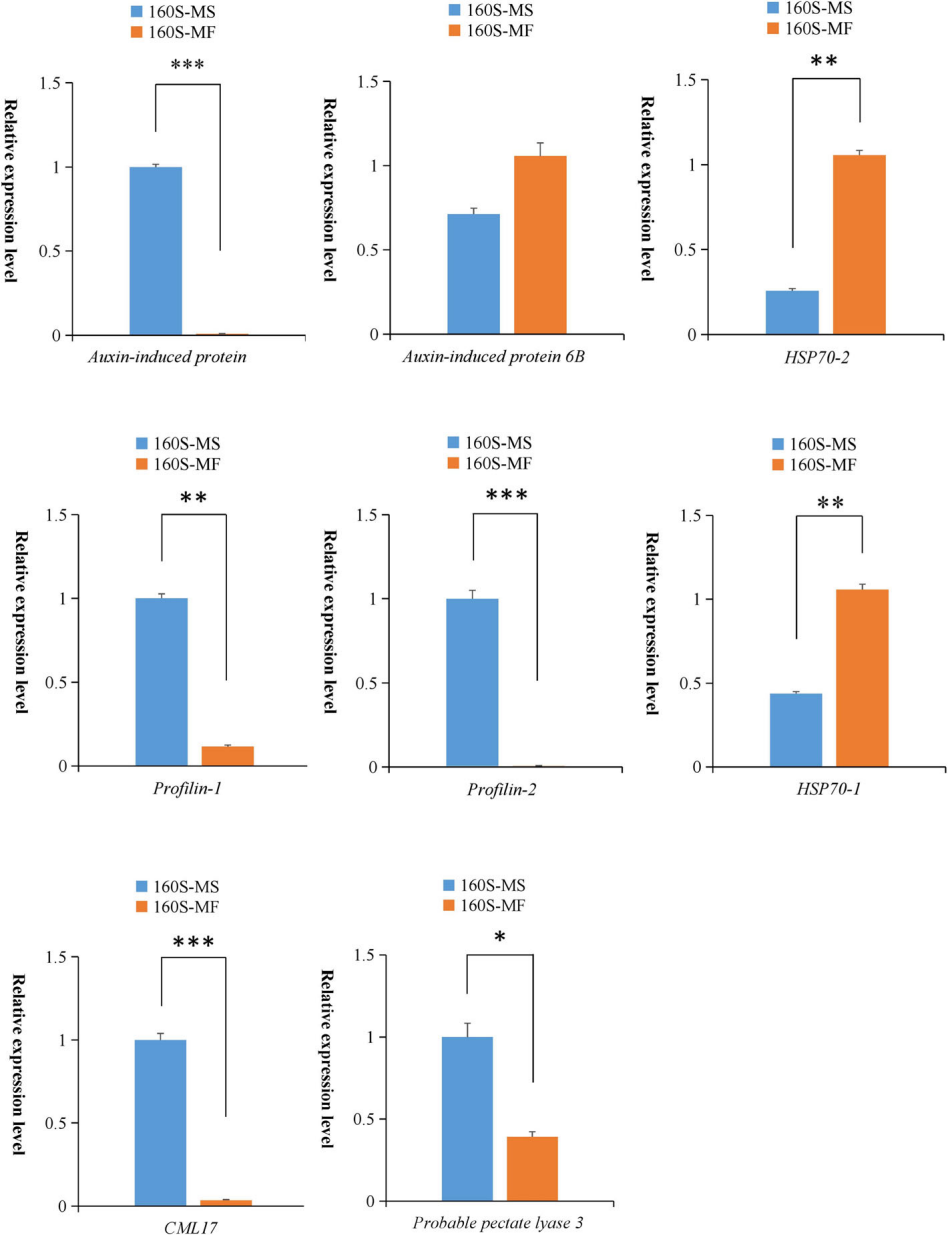
qPCR validation of selected genes. The relative expression levels of unigenes were normalized with internal reference gene actin and 18sRNA. Values are means ± SE with three replicated for each samples in qPCR

To confirm the expression profiling obtained by RNA-seq analysis, the expression levels of 8 genes (encoding auxin-induced protein, auxin-induced protein6B, HSP70–1, HSP70–2, proflin-1, proflin-2, CML17 and probable pectate lyase 3) putatively involved in MS and showing differential expression patterns between 160S-MS and 160S-MF were further validated by qPCR. Our results revealed that the expression level change of all the selected genes based on qPCR analysis agreed with those detected by RNA-seq analysis (Fig. 4). However, the fold changes in expression level of some genes were different depending on the detection method. The transcript levels of *auxin-induce protein* and *proflin-2* were only detected in 160S-MS. *Profilin-1*, *CML17* and *probable pectate lyase 3* were significantly up-regulated in 160S-MS, while *auxin-induced protein 6B*, *HSP70–1* and *HSP70–2* were remarkably up-regulated in 160S-MF.

### Possible molecular mechanisms of male sterility of *B. napus* under the higher temperature

**Fig. 5:**
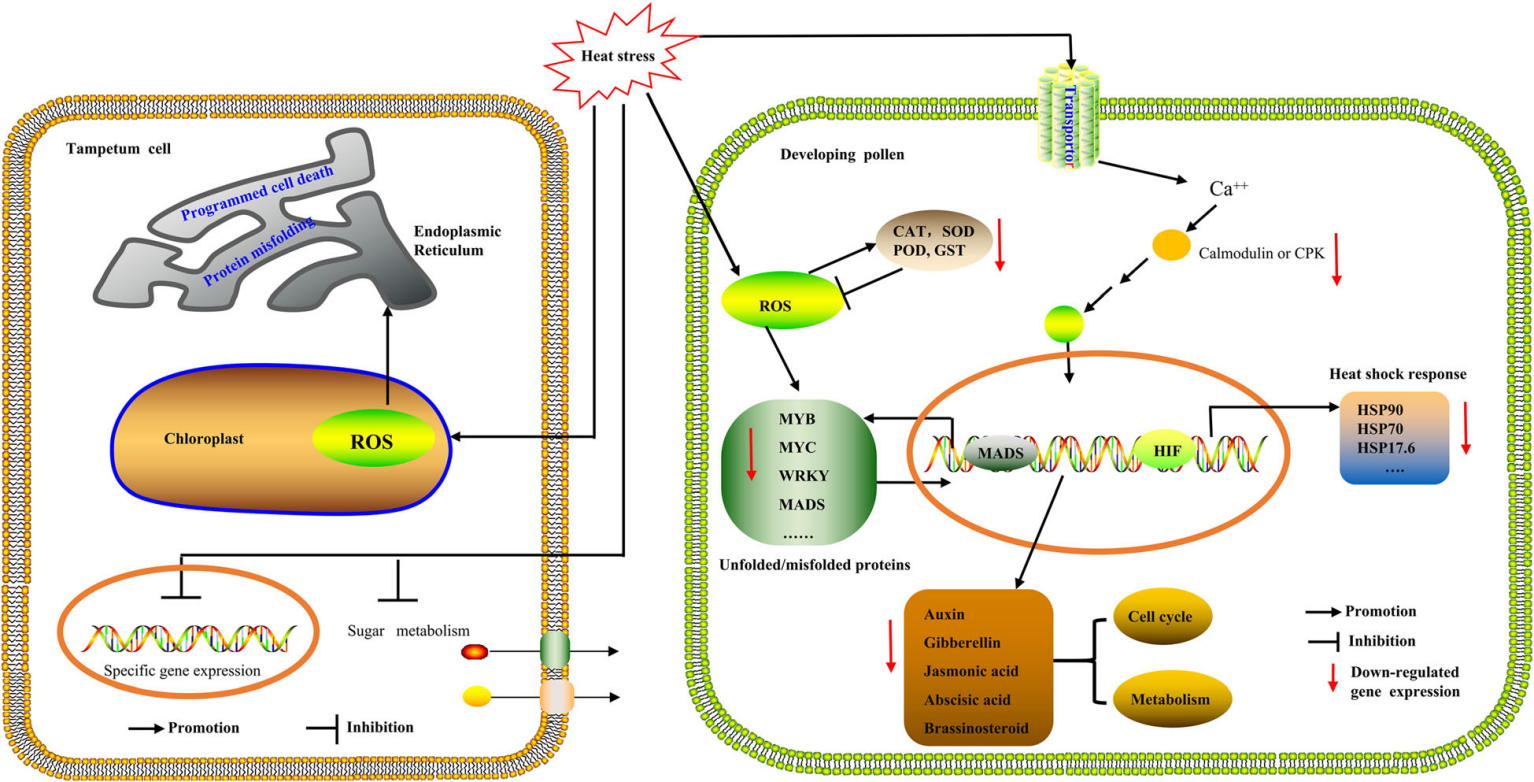
The putative molecular mechanism of male sterility in *Brassica napus*

Developing pollen and the surrounding tapetal cells show a high sensitivity to heat stress that often results in premature degeneration of tapetal cells and aberrant developmental or programmed cell death. While the cause of this sensitivity remains largely unknown, we suggest a schematic model to illustrate the possible roles of some key players in the developing pollen that might be related to TGMS under heat stress (Fig. 5).

### Abiotic stress and sterility

#### Heat shock proteins

Natural floral organ abortion or degeneration results in unisexual or fully sterile flower, while abiotic factors lead to sterility after initiation of floral reproductive organs. Even a mild or short term of abiotic stress can cause a remarkable decrease in fertility. From floral emergence to 3 days post anthesis, heat stress (36 (day)/31 °C (night)) results in male sterility of wheat due to abnormal pollen grains [24]. Barley grown under the high temperature (30–35 °C day/20–25 °C night) displayed tapetum degradation [25]. Rices that receives a short-term (33.7 °C, 1 h) or a long-term high temperature explosure (38 °C, 41 °C, 5 days) displayed significantly lower fertility, but a better fertility was observed when heat stress was applied before or after anthesis [26]. Although the mechanism of the heat-induced sterility is not clear, it might be related to heat shock proteins (HSP). In *Arabidopsis*, disruption of *HSP40* caused pollen tubes to burst and decrease pollen tubes length [27].

Similarly, mutaion in a small heat shock protein gene *BOB1* results in irregular flowers and sterilize siliques of *Arabidopsis* [28]. In maize, pollen sterility may be due to the missing protection of some HSPs [29, 30], supporting the fact that some *HSP* genes did not express at dehiscence [31]. In rice, heat stress induces many *HSP* genes, including *HSP70*, *HSP26* and *HSP17* [32]. Interestingly, six *HSP* genes (*HSP17.6*, 4 *HSP70s* and *HSP90*) were significantly down-regulated in 160S-MS flowers (Additional file 7: Table S6), implying that low transcription level of *HSP* genes may contribute male sterility of *B. napus*. Of course, more work needs to be done to understand the detail role of HSP in male sterility.

#### Antioxidants

Another major mechanism underlining high temperature-induced damages in developing pollen and/or tapetal cells is through the accumulation of reactive oxygen species (ROS), which typically causes membrane degradation, reduced translation and transcription, and eventually programmed cell death (PCD) [33–35]. Plants have evolved an effective non-enzymatic system (e.g. glutathione, ascorbic acid) and enzymatic system (e.g. SOD, APX, CAT, GR and GST) to scavenge the accumulated ROS. In many cytoplasmic male sterile crop varieties (e.g. rice, peper, cotton), pollen PCD is caused by an excessive mitochondrial accumulation of ROS together with a decreased scavenging capacity in the developing spores. Under the high temperature condition (25 °C), decreased expressions of eight antioxidant-related genes (encoding SOD, CAT, GST and POD) were observed in 160S-MS (Additional file 7: Table S6), which was consistent with our previous results that antioxidative enzyme activities were higher 160S-MF [23]. It is possible that high temperature leads to higher respiration rate and excessive ROS is accumulated in 160S-MS flowers, thus low activties of antioxidative enzymes scavenge them defeciencely and result in the pollen abortion.

### Role of hormones in male sterility

Plant hormones have strong influences on flower and fertility. Some hormones are essential for both male and female organ development, while others are male or female specific [36]. To investigate the relationship between the hormone and the male sterility in *B. napus*, the enriched hormone signaling pathways were analyzed (Additional file 7: Table S6).

#### Auxin

Due to the importance of auxin in floral organs formation, disruption of genes associated with auxin biosynthesis, transport and signaling leads to flowers with various abnormalities. In barley and *Arabidopsis* anthers, heat stress (5 day at 30/25 °C day/night in barley and 31–33 °C in *Arabidopsis*) significantly reduced endogenous auxin level in developing pollen mother cells (PMCs) and tapetal cells through suppressing the transcript level of *Yuc2* and*Yuc6* (encoding auxin biosynthesis enzymes) which leads to a premature abortion of microspore development [37, 38]. Auxin response factors (ARFs) repress or activate expression of auxin response genes. In *Abrabidopsis*, the *arf6/arf8* double mutant displays shorten petal and stamen filaments, failure to release pollen, and abnormal ovules [39, 40]. Similarly, double mutation of *arf3(attin)/let* causes a decreased number of stamens [41], while mutation in *arf5/mp* leads to either small or absent lateral flowers [42]. The differential expression of *arf7* in 16S-MS and 160S-MF might be related with male sterility.

#### Gibberellins

Gibberellins (GA) is necessary for filament elongation and pollen development in *Arabidopsis* and *Oryza sativa*. GA signaling functions through the degradation of DELLA proteins, a class of transcriptional repressors that inhibit GA-dependent variations in the expression of down-stream target genes [43, 44]. *DELLA* loss-function mutant displays sterility phenotype due to abnormal microsporogenesis and delayed growth of all floral orangs, e.g. stamens with shortened filament that cannot pollinate pistils [45]. As a key member of GA signaling pathway, four *DELLA* gene was significantly up-regulated in 160S-MS flowers (Additional file 7: Table S6), highlighting that GA is required for male sterility in *B. napus*. Another analysis showed that DELLA proteins could suppress the synthesis of JA that is required for the activation of transcription factor MYB21/24/57, which is essential for late stamen filament growth in *Arabdopsis* [46].

#### Jasmonic acid

Jasmonic acid (JA) has been known as a growth regulator acting as a signal for plant growth, development and fertility. JA signaling is activated jasmonate-ZIM domain proteins (JAZ) that binds to and suppress the activities of transcription factors, such as MYC family, that regulating the expression of JA-responsive genes [47, 48]. In *Arabidopsis* and maize tassel, JA is also identified as a factor affecting anther dehiscence, stamen and pollen maturation [49, 50] .The *opr* mutant is deficient in JA synthesis and produces staments with abnormal filament elongation and dehiscence in *Arabidopsis* [51]. Silimarly, dobule mutation of *opr7*/*opr8* in maize forms feminized tassel devoid of stamen formation, and extreme elongation of ear shanks [52]. Jasmonate acid carboxyl methyltransferase (JMT) is a key enzyme responsible of convertion of JA to MeJA. The down-expression of *JMT* and JAZ genes in 160S-MS flowers (Additional file 7: Table S6) would result in a decreased JA level. This deficiency might contribute male sterility in *B. napus*. Further study looking into the roles of these enzymes on sterility could be valuable.

#### Abscisic acid

As a key hormone involved in abiotic stress resposnes, abscisic acid (ABA) plays an important role in male fertility during reproductive stress [53]. In rice, ABA regulates the expression of tapetum cell wall bound invertase and monosaccharide transport genes, leading to a disturbed carbohydrate accumulation in the anther and thus, in pollen sterility [54]. Regulatory component of ABA receptor PYR/PYL senses ABA and initiates the ABA signaling. The expression level of two *PYR/PYL* genes were significantly decreased in 160S-MS (Additional file 7: Table S6), suggesting that the lack of ABA signal might be involved in *B. napus* sterility*.* Because both ABA biosynthesis and catabolism afftect ABA signaling in plant tissues, therefore further studies focusing on the mechanism that control ABA homeostasis during the reproductive stages would provide useful information for male sterility study.

#### Brassinosteroid

Brassinosteroids (BRs) are perceived by a plasma membrane-localized receptor, brassinosteroid insensitive 1(BRI1), and essential for male fertility in plants. In *Arabidopsis*, BR-deficient cpd mutant displays male sterility due to the failure of pollen tube elongation. Similarly, mutation of dwf4 results in reduced filament elongation, leading to a failure of pollen delivery to the stigma [55, 56]. However, the underlying mechanism of BRs in regulating anther/pollen development is completely unknown. As the receptor of BR, two *BRI1* genes was remarkably down-regulated in 160S-MS flowers (Additional file 7: Table S6), implying that BR controls male sterility at least in part via directly regulating key genes for anther and/or pollen development in *B. napus.*

### Molecular genetic regulation of male sterility

Many transcription factors (TFs) are well known to control plant growth and development and several TFs have been reported to be specially related to anthers and pollen development [57]. In this study, genes encoding TFs, such as MADS-box transcription factor (MADS), nuclear transcription factor Y(NFY), heat shock transcription factor (HSF), MYB/C and WRKY were identified and deferentially expressed in 160S-MS flowers and 160S-MF flowers (Additional file 7: Table S6). The MADS-box genes encode a family of TFs that control diverse developmental processes such as meristem identity, flowering time determination, floral organ identity and silique morphogenesis [58]. In rice and Arabidopsis, MIKC type MADS-box genes was expressed in the gametophyte, while MADS62, MADS63 and MADS68 were all specifically expressed late in pollen development and their reduced expression result in defective anther development and degenerated pollen grains. Knock-down of MADS2 resulted in anther abortion and pollen development defect in maize [59]. In *Oryza sativa*, MADS3 is required for stamen identity determination during early flower development and also played a critical role in late anther development by modulating ROS homeostasis [60]. Nine MADS genes showed significantly lower expression in sterile flower, which suggested that them may be involved in floral organ identity or other functions during anther development. The nuclear factor Y(NFY) transcription factors are considered as important regulators of many plant developmental processes, including tolerance/resistance to abiotic stresses and male sterility [58]. Two α-subunit genes (NFYA) were down-regulated in sterile flowers, indicating that they might be involved in thermo-response during the anther development.

The MYB superfamily is characterized by a conserved DNA-binding domain, the MYB domain, and is one of the largest TF families in plants. Some MYB transcription factors have been found to play important role in flower development. In *Arabidopsis thaliana*, disruption of AtMYB26 results in male sterility in due to non-dehiscent anthers [61]. AtMYB103 is especially expressed in anthers and its decreased expression results in early degeneration of the tapetum and abnormal pollen grains [61]. AtMYB24 is induced by jasmonates and is essential for filament elongation [59]. AtMYB80 regulates programmed cell death of the tapetum in the anther, and thus required for pollen development [62]. However, it is not known whether any other MYB transcription factor also plays an essential role in anther, stamen and pollen development, pollen tube growth, especially in agriculture important crops. In this study, the transcript levels of two MYB genes were increased in sterile flowers, but 17 MYB genes were down-regulated. Further analysis will be performed to determine whether some of them participates the regulation of the male sterility of *B. napus*.

### Other miscellaneous molecules putatively involved in male sterility

#### Skeleton proteins

The actin cytoskeleton is known to play key roles in the morphogenesis and function of highly specialized cell types. Aberrant regulation of actin- and tubulin-related genes can disrupt the organization of actin and microtubules in meiotic process, which leads to defective cytokinesis in developing pollens and male sterility [63]. It has been suggested that low actin levels in anthers are associated with the male sterility in some plants [64]. In the present study, 17 actin-related genes were up-regulated and one β-tubulin-related gene was down-regulated in the flowers of 160S-MS (Additional file 7: Table S6). Actin depolymerizing factor (ADF), an actin binding protein, plays a role in regulating F-actin filament assembly. In *Arabidopsis*, the overexpression of *AtADF1* resulted in the disappearance of thick actin cables in different cell types and caused irregular cellular and tissue morphogenesis, and development arrest. In contrast, the down-regulation of *AtADF* could promote the formation of actin cables and resulted in a delay in the flowering and organ growth [65]. High expression of *TaADF* (4.28- fold change) was also found in SQ-1 treated wheat sterile line compared with the fertilize line, which caused the microfilament desambly and the accumulation of callose [66]. In the present study, 7 actin depolymerization factor/cofilin-like domains (ADF domains) genes were up-regulated in 160S-MF flowers at 25 °C, suggesting that the defective ckeleton dynamics in the anther might be associated with the male sterile.

#### Rho of plant (ROP)

ROP proteins are members of GTPases and function in many fundamental cellular activities, such as cell polarity establishment in pollen tube, cell morphogenesis, regulation of actin cytoskeleton, and hormonal response, and also been implicated in abiotic and biotic stress response [67–69] . In *Arabidopsis*, a mutation in *SPIKE1*, coding for a ROP protein, leads to the stunted growth and severe defects in polarized cell growth [70]. Additionally, the epidermal pavement cell morphogenesis is regulated by the counter signaling of two Rop-mediated antagonistic pathways, the coordination between plant hormone auxin and specific Rop GTPases together organizes and restructures the cytoskeletal elements for cell morphogenesis and patterning [71]. We found that the transcript levels of 3 *ROP* genes were significantly increased in 160S-MS flowers (Additional file 7: Table S6), suggesting that they might be associated with the thermo-sensitive male sterility. Till now, only a few ROPs and their interacting proteins have been characterized and more detailed studies will be needed to generate further insights into the related signaling pathways in TGMS.

#### Calcium signal

In plant, calcium-dependent protein kinase (CPK)-mediated signal has been implicated in many biological processes, such as environmental stress response, pollen tube growth and fertilization [72]. In maize, several CPKs were enriched in pollen and might serve as an essential switch in regulating maize pollen tube growth [73]. For example, *ZmCPK32* is specifically accumulated in pollen during shedding and putatively associated with pollen tube development [74]. Despite numerous reports on the role of calcium and calmodulin (CaM) in pollen germination and tube growth, the proteins that mediate calcium/CaM action have not been identified in *B.napus*. In this study, the expression levels of 10 *CaM* genes (2 *CML3*, 2 *CML17*, 2 *CML25*, *CML7*, *CML42*, *Polcalcin Bra r1* and *2*) were up-regulated, while 2 genes (*CML21*, *CML35*) were down-regulated in 160S-MS flowers (Additional file 7: Table S6), which supported the mechanism that calcium signaling pathway is responsible for male fertility.

## Conclusion

In this study, 2202 genes were significantly differentially expressed in the flowers at 25 °C compared with these at 15 °C. Functional enrichment analysis revealed that genes encoding heat shock proteins, antioxidant, skeleton protein, and calmodulin might be involved in TGMS of *B. napus*. Interestingly, the transcription levels of some key members in signaling pathways, intermediated by auxin, gibberellins, jasmonic acid, abscisic acid, brassinosteroid signalings, were significantly surppressed in sterile flowers. Moreover, well-known transcription factor MADS, HSF, MYB/C and WRKY might be involved in male fertility under the high temperature. Our results are helpful for understanding the molecular mechanisms of *B. napus* TGMS and also provide an effective foundation for male fertility researches in other important economic crops.

## Methods

### Plant materials

*Brassica napus* 160S were grown at 15 ± 1 °C under long day length before the bud forming, and then moved into the climate chambers at 25 ± 1 °C with a photoperiod of 14 h light and 10 h dark, and 65% relative humidity (RH) until the sterile flower forming. The naturally growing young sterile flowers were collected, frozen in liquid nitrogen, and stored at − 80 °C for later use.

### Illumina sequencing and de novo assembly

Total RNA from the sterile or fertile flowers was extracted using the Trizol reagent (Invitrogen, USA) and mRNA was purified using the RNeasy Plant Mini Kit (Qiagen, Valencia, CA). RNA concertrations were measured using the NanoDrop 2000 spectrophotometer (Thermo Fisher Scientific, USA) and the integrity was determined using a Bioanalyzer 2100 (Aligent, CA). The RNA integrity number (RIN) values were greater than 8.5 for all samples. Sequencing libraries were prepared according to the manufacturer’s instructions (Illumina, CA). mRNA was subjected to two rounds purification using poly(T) oligo-attached magnetic beads, and fragmented using an RNA fragmentation kit. First strand cDNA was generated using reverse transcriptase and random primers. Following the second strand cDNA synthesis and adaptor ligation, 200 bp cDNA fragments were isolated using gel electrophoresis and amplified by 18 cycles of PCR. The products were loaded onto an Illumina HiSeq2000 instrument and subjected to paired-end sequencing. Images analysis, base-calling, raw tags generation, and tags counting were performed using the Illumina data processing pipeline.

### Sequence annotation

Raw reads were trimmed to obtain high-quality reads by removing adaptors and low-quality tags. All clean reads were assembled and annotated against the following database: Nt (NCBI non-redundant necleotide sequences), Nr (NCBI non-redundant protein sequences), Pfam (Protein family), UniProtKB/Swiss-port (the UniProt Knowledgebase), KOG (euKaryotic Ortholog Groups), GO (Gene Ontology); and KO (KEGG Ortholog database). All BLAST searches were performed with the cut-off of E-value ≤1E^− 5^.

### Differential gene expression, GO and KEGG enrichment analysis

To identify the differentially expressed genes (DEGs) between two samples, the number of expressed tags was calculated and then normalized in terms of RPKM (Mortazavi A, 2008). Genes with a absolute value of ∣log_2_^ratio^∣ ≥ 1, False discovery rate (FDR) ≤ 0.05 and the adjusted *P*-value < 0.05 were assigned as differentially expressed. Differentially expressed transcripts were classified into functional categories using Blast2GO with default parameters [75], based on three levels of GO terms: biological processes (BP), molecular function (MF) and cell components (CC). GO and KEGG enrichment analysis of DEGs was performed using the GOseq R packages (http://www.r-project.org/) and KOBAS (http://www.kobas. co.uk/), respectively.

### Validation of DEGs by qPCR

To verify the accuracy of RNA-seq results, 8 DEGs were selected for relative qPCR analysis. Total RNA was extracted, treated with DNase, and was reverse transcribed into cDNA using a RevertAid First Strand cDNA Synthesis kit (Fermentas, Vilnius, Lithuania). SYBR-based qPCR reactions were performed on a LightCycler 480 system (Roche, Switzerland) with the following reaction conditions: 95 °C for 1 min, followed by 40 cycles of 95 °C for 10 s, and 60 °C for 30 s. All PCR reactions were performed in triplicate, and the relative expression levels were calculated with the 2^-ΔΔCT^ method [76] using *Ubc9* and *Ubc21* as the references. Gene-specific primers were listed in Additional file 8: Table S7.

## Additional files

Additional file 1: **Table S1.** Sumary of the RNA-seq data. (XLS 19 kb)

Additional file 2: **Figure S1.** Gene annotation statistics (A) and species classification (B). (PNG 1091 kb)

Additional file 3: **Table S2.** Gene Ontology (GO) terms assigned to the unigenes. (XLSX 11 kb)

Additional file 4: **Table S3.** KOG anotation of the mapped genes. (XLSX 9 kb)

Additional file 5: **Table S4.** Functional annotation of DEGs based on KEGG categorization. (XLSX 20 kb)

Additional file 6: **Table S5.** KEGG pathway enrichment analysis of differentially expressed genes between 160S-MF and 160S-MS. (XLS 50 kb)

Additional file 7: **Table S6.** Differentially expressed genes putatively involved in TGMS of *B. napus. (XLS 36 kb)*

Additional file 8: **Table S7.** Primers used for qRT-PCR. (XLSX 9 kb)

## Data Availability

The sequence raw data from this study have been submitted to the NCBI Sequence Read Archive (SRR8398869) (https://www.ncbi.nlm.nih.gov/sra/?term=SRR8398869).

## Abbreviations

ABA: Phytohormone abscisic acid
ADF: Actin depolymerizing factor
AIP: Auxin-induced protein
APX: Ascorbate peroxidase
ARF: Auxin response factor
ARG: Indole-3-acetic acid-induced protein
AsA: Ascorbic acid
AUX: Auxin influx carrier
BR: Brassinosteroid
CaM: Calcium and calmodulin
CAT: Catalase
CG: Chemical gametocide
CMS: Cytoplasmic male sterility
CPK: Calcium-dependent protein kinase
DEGs: Differentially expressed genes
GA: Gibberellins
GH3: Indole-3-acetic acid-amido synthetase
GMS: Genic male sterility
GSH: Glutathione
HSF: Heat shock transcription factor
HSP: Heat shock proteins
JMT: Jasmonate acid carboxyl methyltransferase
KEGG: Kyoto encyclopedia of genes and genomes
KOG: Eukaryotic orthologous groups
MADS: MADS-box transcription factor
NFY: Nuclear transcription factor Y
PCD: Programmed cell death
POD: Peroxidase
ROP: Rho of plant
SI: Self incompatibility
SOD: Superoxide dismutase
TFs: Transcription factors
TGMS: Thermo-sensitive genic male sterility
