# Supplementary figures and images for: Transcriptomic analysis reveals the mechanism of thermosensitive genic male sterility (TGMS) of Brassica napus under the high temperature inducement
Source: BMC Genomics. 2019 Aug 13;20:644. doi: 10.1186/s12864-019-6008-3 (PMC6691554; doi:10.1186/s12864-019-6008-3)

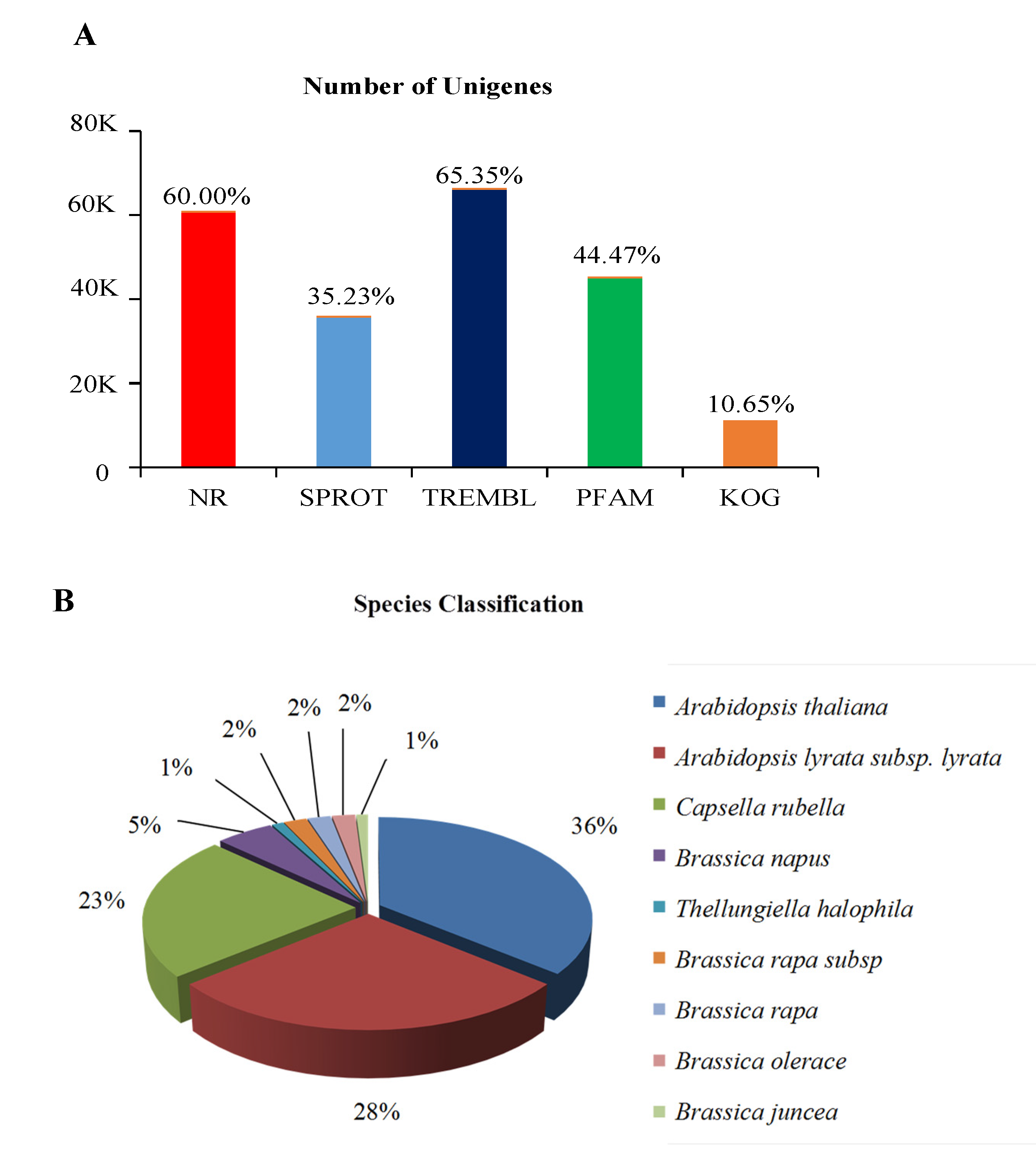

Supplement: Supplementary file 2 — Figure S1. Gene annotation statistics (A) and species classification (B). (PNG 1091 kb) [file 12864_2019_6008_MOESM2_ESM.png]
